# Serum Zinc Concentrations Correlate with Mental and Physical Status of Nursing Home Residents

**DOI:** 10.1371/journal.pone.0117257

**Published:** 2015-01-30

**Authors:** Renata Markiewicz-Żukowska, Anna Gutowska, Maria H. Borawska

**Affiliations:** 1 Department of Bromatology, Medical University of Bialystok, Bialystok, Poland; 2 Nursing Home in Bialystok, Swierkowa 9, Bialystok, Poland; TNO, NETHERLANDS

## Abstract

**Introduction:**

Zinc (Zn) is one of the most important trace elements in the body. Zn deficiency seems to play a role in the development of age-related diseases and impairment of quality of life. Zn status has been especially studied in free-living or hospitalised people, but data from older residents of nursing homes are scarce. This study aimed to determine the Zn status among the older individuals in correlation to their mental and physical performance.

**Methods:**

A total of 100 participants aged between 60-102 years were recruited between October 2010 and May 2012 at the nursing home in Bialystok (Poland). Zn status was evaluated by determining the concentration in serum by flame atomic absorption spectrometry. Anthropometric variables and fitness score (FS) were measured. Abbreviated Mental Test Score (AMTS), Geriatric Depression Scale (GDS), Self-Rated Health (SRH), independence in Activities of Daily Living (ADL) were recorded.

**Results and Discussion:**

The mean serum Zn concentration was 0.83±0.20 mg/L, 28% of residents had Zn deficiency. Cognitive functions were impaired (AMTS≤8) in 45% of the studied persons and 48% showed depressive symptoms (GDS≥1). The ability to independently perform activities of daily living (ADL = 6) was found in 61% of participants, but most of them (90%) had weak body type (FS<70), correlating with GDS, SRH and body mass index (BMI). Serum Zn concentration correlated with mental efficiency and was statistically significantly higher in older people with normal cognitive function and without depression than in patients with memory impairment and showing depressive symptoms.

**Conclusions:**

Nursing home residents seem at risk of marginal Zn status, which correlates with their mental status as measured by the AMTS and GDS. Their low FS is associated with mental health deterioration and obesity.

## Introduction

The growing percentage of older people in the world indicates an increasing number of individuals with serious health problems, functional disability and multiple diseases. In addition to many somatic symptoms and chronic diseases of old age, mental disorders are a common problem—these include cognitive impairment, syndromes of dementia and depression, often unrecognized. Since depression is a common health problem in old age, with prevalence rates of up to 46% in institutionalized older people, there is a need for early diagnosis and treatment, which may contribute to improving their quality of life [1–3].

An increased interest is observed in biomarkers of aging and distinct age-related changes such as disabilities, physical function decline, frailty, diseases of old age, and mortality [4, 5]. A decline in physical performance and function often marks the early stage of the ageing process [6]. Therefore, there is a need to identify those biomarkers related to the age-associated decline in mental and physical performance. It has been shown that low serum micronutrient concentrations are an independent risk factor for frailty among disabled older women, and the risk of frailty increases with the number of micronutrient deficiencies [7].

Nutritional deficiencies resulting in an increased incidence of age-related pathologies are well documented in ageing individuals [8]. Numerous clinical and epidemiological data focused on the importance of nutritional micronutrients status in maintaining performance capacities and quality of life in ageing population [9]. Since Zn affects many age-related functions [10], it is essential to maintain or restore an optimal Zn status in older people. The available studies on Zn status describe free-living or hospitalised people and focus on inadequate Zn intake as well as on relation to humoral immune function in ageing [11, 12].

Zinc in the synaptic vesicles of neurons plays a role in brain activities like learning and memory function. Zn acts as a neurotransmitter [13, 14]. It has been shown that Zn deficiency is associated with certain problems commonly seen in the older population, such as frequent infection episodes, loss of taste and appetite, difficulty seeing at night, defect in bone mineralization, hair loss, depression, difficulty in concentration and mental lethargy [10, 15].

There is a limited number of published reports on the relationship between Zn status and mental and physical health among nursing home residents. Therefore, the aim of the present study was to examine the Zn status in a nursing home population and to determine whether Zn serum concentration is related to their mental and physical performance.

## Methods

### Participants

The study was performed between October 2010 and May 2012. All of the 130 residents living in the nursing home (Bialystok, Poland) were invited to participate in the study. A total of 30 residents or their legal representatives, either declined to participate or it was not possible to contact them. All study participants were informed about the objectives of the study and the written consent was obtained from each participant or their legally authorized representative. The study protocol was approved by the Local Bioethical Committee of Medical University of Bialystok (UMB-R-I-002/259/2008). All participants completed a study questionnaire with assistance of the researchers during face-to-face interviews. The blood drawing was done on the same day as the tests. Fasting blood samples were collected in the Vacutainer Systems tubes containing clot activator (Becton Dickinson, France). The blood was centrifuged. The serum was removed and kept frozen at -80°C.

Data on age, number of diagnosed diseases and dosages of prescribed medications were collected from the residents’ files by a physician. The participants took only the medications prescribed by their medical doctor and they did not take any supplements. Anthropometric variables included height and weight measured to the nearest 0.1 cm and 0.1 kg, respectively, using height-measuring equipment connected with an electronic scale (AXIS B150L, Seca, Gdańsk, Poland) while the subjects were wearing light-weight clothes. Then, body mass index (BMI) was computed as the ratio of weight (kg) to height squared (m^2^). BMI was used to assess the prevalence of overweight (25–29.9 kg/m^2^) and obesity (≥30 kg/m^2^) according to WHO criteria [16].

We used the bioelectrical impedance analysis system (InBody720, Biospace, Seoul, Korea) to evaluate the mass of muscle and fat tissue in the body composition of participants and based on the results we determined their fitness score (FS). A value of less than 70 points indicated a weak body type, a range of 70 to 90 points—normal body type and greater than 90 points—athletic body type.

### Serum zinc determination

Zn status was evaluated by determining the serum Zn concentration using flame atomic absorption spectrometry on Z-2000 instrument (Hitachi, Japan) with Zeeman background correction [17]. All samples were run in duplicate. Serum concentration <0.7 mg/L was regarded as Zn deficiency [18].

The method of Zn determination was verified using certified reference material (Seronorm Trace Elements Serum L-1, 0903106, Sero AS, Norway). The accuracy (expressed as a percentage of the error) was 1.96% and coefficient of variance (indicator of the precision of this method) was 2.56%. These values did not exceed 2% and 5%, respectively. Moreover, our Department has participated in a quality control program for trace elements’ analysis supervised by the Institute of Nuclear Chemistry and Technology and the National Institute of Public Health (Warsaw, Poland) and our results of the quality control analyses were consistently categorized as being in agreement with reference values.

### Mental state

Cognitive impairment among the study participants was evaluated using the Abbreviated Mental Test Score (AMTS), a brief 10-item survey. Each question answered correctly scored one point and a score of 8 or less suggested cognitive impairment at the time of testing. AMTS was introduced by Hodkinson in 1972 [19]. It is a rapid tool for assessing cognitive function and it is especially useful in determining the risk of dementia in older patients. With time, AMTS has become more widely used in health care, e.g. to assess confusion or cognitive impairment and has been mainly validated in older subjects. Even though it is similar to the Mini Mental State Examination (MMSE), the AMTS does not require reading, writing or drawing and it does not strictly depend on the educational level; overall, a strong linear relationship has been demonstrated between the MMSE and AMTS [20]. A meta-analysis of studies comparing the AMTS (cut-off value of <8) with dementia as defined by the reference standard, showed that the AMTS allows 94% sensitivity and 74% specificity for the diagnosis of dementia [21]. According to the meta-analysis, AMTS is a proper screening instrument.

Emotional status of the study population was examined using the Geriatric Depression Scale (GDS) validated in nursing home residents [22]. We decided to use the short version GDS with only 4 ‘Yes/No’ questions. A score of 1 point or above indicates depression. The short version GDS is not only convenient and saving resources, but it is also more acceptable to older people and can be used in lieu of the longer versions [23, 24].

Self-Rated Health (SRH) was assessed using a 5-point Likert scale (from 1 to 5) with questions pertaining to the respondent’s perceived overall health: “In general, would you say your health is: very poor, poor, almost good, good or excellent?” [25, 26].

### Physical function

Changes in the functional status of older persons are common, multicausal, and can result from a variety of diseases. The Katz index of independence in Activities of Daily Living (ADL) is the most appropriate instrument to assess functional status. The index ranks the adequacy of performing six functions i.e. bathing, dressing, toileting, transferring, continence and feeding. Patients are scored ‘Yes/No’ for independence in each of the functions. A score of 6 indicates full function, 4 indicates moderate impairment, and 2 or less indicates severe functional impairment [27–29].

### Statistical analyses

Statistical analyses were performed using Statistica software version 10.0 PL for Windows (StatSoft, Cracow, Poland). Metric data were tested for normal distribution by the Kolmogorov-Smirnov and the Shapiro-Wilk tests and visual inspection of the Quantile-Quantile (Q-Q) plots. Results are given as mean and standard deviation (SD) and as median and interquartile range (IQR). Normally and non-normally distributed variables of two independent groups were compared using the Student’s t-test and the Mann-Whitney U test, respectively. Correlations between the parameters were described by the Spearman’s rank correlation coefficients. To compare the qualitative variables we used the chi-square test for independence. Analysis of variance was performed using ANOVA tests and post-hoc NIR Fisher’s test. We assessed the effects of the analysed parameters on the variability of Zn concentration using backward multiple regression. A p-value of less than 0.05 was considered statistically significant.

## Results

Patients characteristics and their test results are presented in Table 1. The correlations between the characteristics and test results are given in Table 2. The mean age of the study participants was 76±11 years (range: 60–102), women (79.5±9.8 years) were older (p<0.001) than men (72.2±10.5 years). Overweight or obesity were found in 64% of the examined nursing home residents. In addition, BMI correlated with FS (r = -0.48, p<0.001), with SRH (r = -0.24, p = 0.014) and with GDS (r = 0.20, p = 0.047). Out of all study participants, 64% had 3 or more diseases diagnosed and 95% had been taking at least one medication. The number of diseases showed weak but positive correlations with age (r = 0.21, p = 0.038) and with BMI (r = 0.22, p = 0.028), and negative correlations with FS (r = -0.30, p = 0.002) and SRH (r = -0.39, p<0.001). Similarly, the number of prescribed medications positively correlated with age (r = 0.20, p = 0.044) and BMI (r = 0.22, p = 0.025), and negatively with FS (r = -0.21, p = 0.046) and AMTS (r = -0.20, p = 0.041).

**Table 1:** Characteristics of the study population.

Variable	n	Median (IQR)	Mean ± SD (range)	(%)
**Age** (years)	100	77 (69–84)	76 ± 11 (60–102)	
≥80	37			37
**Sex** (M/F %)	100			48/52
**BMI** (kg/m^2^)	100	27.5	27.5 ± 6.5	
		(23.0–30.9)	(15.3–61.8)	
<18.5	4			4
18.5–24.9	32			32
25–29.9	33			33
≥30	31			31
Number of diseases	100	3 (2–4)	3.3 ± 1.7 (1–8)	
≥3	64			64
Number of medications	100	4 (3–6)	4.4 ± 2.5 (0–11)	
≥ 1	96			96
**Zinc** (mg/L)	100	0.79	0.83 ± 0.20	
		(0.69–0.98)	(0.43–1.39)	
<0.7	28			28
**AMTS** (points)	100	9 (5.5–10)	7.4 ± 3.2 (0–10)	
≤8	45			45
**GDS** (points)	100	0 (0–1)	0.9 ± 1.1 (0–4)	
≥1	48			48
**SRH** (points)	100	3 (3–4)	3.2 ± 0.9 (1–5)	
<3	20			20
**ADL** (points)	100	6 (5–6)	5.1 ± 1.5 (0–6)	
<6	39			39
**FS** (points)	100	61 (55–66)	60 ± 7 (42–72)	
<70	90			90

n—number of subjects, SD—standard deviation, IQR—interquartile range, M—male, F—female, BMI—Body Mass Index, AMTS—Abbreviated Mental Test Score, GDS—Geriatric Depression Scale, SRH—Self-Rated Health, ADL—independence in Activities of Daily Living, FS—fitness score.

**Table 2:** Spearman’s rank correlation coefficients between measured parameters (and p values for significant correlation).

Parameters	Zn	Age	BMI	No of diseases	No of medications	AMTS	GDS	SRH	ADL
Age	**-0.28** (0.005)								
BMI	-0.15	0.01							
No of diseases	-0.15	**0.21** (0.038)	**0.22** (0.028)						
No of medications	-0.10	**0.20** (0.044)	**0.22** (0.025)	**0.51** (<0.001)					
AMTS	**0.38** (<0.001)	**-0.39** (<0.001)	-0.14	-0.16	**-0.20** (0.041)				
GDS	**-0.28** (0.006)	0.13	**0.20** (0.047)	0.14	0.06	-0.08			
SRH	0.18	-0.15	**-0.24** (0.014)	**-0.39** (<0.001)	-0.15	**0.22** (0.029)	-0.17		
ADL	0.07	-0.10	-0.02	-0.18	-0.18	**0.26** (0.009)	-0.09	**0.30** (0.002)	
FS	0.19	-0.19	**-0.48** (<0.001)	**-0.30** (0.002)	**-0.20** (0.046)	0.18	**-0.50** (<0.001)	**0.36** (<0.001)	0.19

BMI—Body Mass Index, AMTS—Abbreviated Mental Test Score, GDS—Geriatric Depression Scale, SRH—Self-Rated Health, ADL—independence in Activities of Daily Living, FS—fitness score.

Correlation coefficients values between 0 and 0.3 (0 and—0.3) indicate weak; between 0.3 and 0.7—moderate; between 0.7 and 1.0 strong positive (negative) relationship.

**Fig 1:**
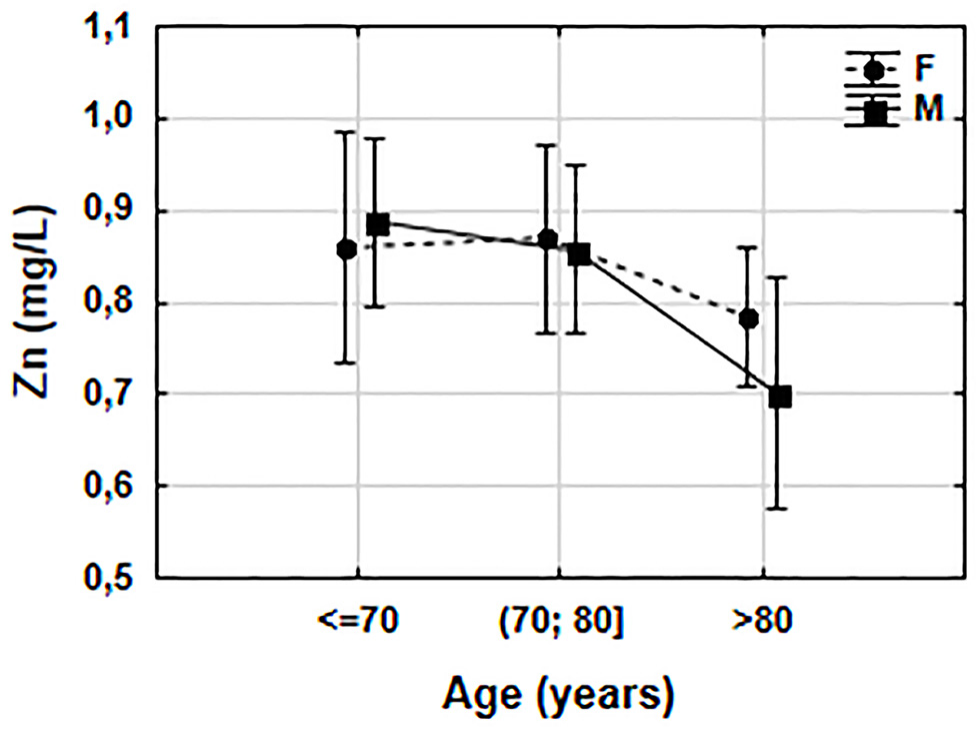
Influence of age and gender on zinc status. The results are obtained in ANOVA test. F(2, 94) = 0.5479, p = 0.580. Vertical bars represent the 0.95 confidence intervals. F-female, M-male.

Serum Zn concentrations in our subjects ranged from 0.43 mg/L to 1.39 mg/L. Categorization of Zn concentration showed proper Zn status in most individuals—the prevalence of Zn deficiency was 28%. The change in the Zn status was associated with age (r = -0.28, p = 0.005) and with mental health parameters: AMTS (r = 0.38, p<0.001) and GDS (r = -0.28, p = 0.006). Chi-square test for independence (Table 3) also indicated an association of Zn status with age (χ2 = 11.32, df = 4, p = 0.023). We determined that Zn concentration was below normal in only 17% of the participants aged ≤70 years, and in 43% of those aged >80 years. The ANOVA test showed that gender did not influence the Zn status and no interaction between age and gender was found. Age affected the serum Zn concentration in both men and women, particularly in the oldest age. This trend was clearly marked for men (Fig. 1). Post-hoc NIR Fisher’s test underlined lower serum Zn concentration in the oldest participants (>80 years) compared to those below 70 years of age (p = 0.021) and those aged 70–80 years (p = 0.037).

**Table 3:** Measured parameters according to zinc status evaluated by serum zinc (Zn mg/L) concentration.

Parameters (n)		Percent of people			**p** (χ^2^, d.f.)
		Zn <0.7	Zn: 0.7–1.2	Zn >1.2	
**Age** (years)	≤70 (29)	17	76	7	0.023* (11.32, 4)
	71–80 (34)	21	65	15	
	>80 (37)	43	57	0	
Sex	M (48)	27	65	8	0.876 (0.26, 2)
	F (52)	29	65	6	
**BMI** (kg/m^2^)	<18.5 (4)	50	50	0	0.277 (7.49, 6)
	18.5–24.9 (32)	19	72	9	
	25–29.9 (33)	24	64	12	
	≥30 (31)	39	61	0	
No of diseases	1–2 (34)	26	62	12	0.497 (3.37, 4)
	3 (28)	21	71	7	
	≥4 (38)	34	63	3	
No of medications	0–2 (23)	17	78	4	0.675 (4.01, 6)
	3–5 (47)	30	62	8	
	6–8 (24)	33	58	8	
	≥9 (6)	33	67	0	
**AMTS** (points)	≤8 (45)	42	56	2	**0.008** * (9.70, 2)
	>8 (55)	16	73	11	
**GDS** (points)	= 0 (51)	20	69	12	**0.045** * (6.20, 2)
	≥1 (49)	37	61	2	
**SRH** (points)	<3 (20)	30	70	0	0.390 (1.88, 2)
	≥3 (80)	27	64	9	
**ADL** (points)	<6	31	61	8	0.845 (0.34, 2)
	= 6	26	67	7	
**FS** (points)	<70	29	64	7	0.803 (0.44, 2)
	≥70	20	70	10	

n—number of subjects, p-value for χ^2^test for independence, χ^2^—chi-square, d.f.—degrees of freedom, M-male, F-female, BMI—Body Mass Index, AMTS—Abbreviated Mental Test Score, GDS- Geriatric Depression Scale, SRH—Self-Rated Health, ADL—independence in Activities of Daily Living, FS—fitness score.

*Significant differences

The normal cognitive functions (AMTS>8) were found in 55% of the studied individuals (Table 1). The prevalence rate of well-being (SRH≥3) was 89% in the group with the normal cognitive functions and 69% in patients with impaired cognitive functions, which showed a significant difference (χ2 = 6.31, df = 1, p = 0.012). The difference in the prevalence rate of ADL = 6 was also significant (χ2 = 12.13, df = 1, p<0.001) in these groups—76% and 42%, respectively. Additionally, we found a moderate negative relationship between AMTS and age (Table 2).

Over a half of the examined participants (52%) showed no signs of depression (Table 1). Among the 48 residents considered to have depressive symptoms according to the GDS, only 3 had a documented diagnosis of depression and 14 had mood disorders in their medical charts. Moreover, the FS was higher (p<0.001) in patients with GDS = 0 vs. patients with signs of depression and additionally a positive correlation was found between SRH and FS (r = 0.36, p<0.001).

The majority of patients had a weak body type (FS below 70 points). The ability to independently perform activities of daily living (ADL = 6) was found in 61% of participants. We observed a moderate positive correlation of ADL with SRH (r = 0.30, p = 0.002) and a weak positive correlation with AMTS (r = 0.26, p = 0.009).

**Fig 2:**
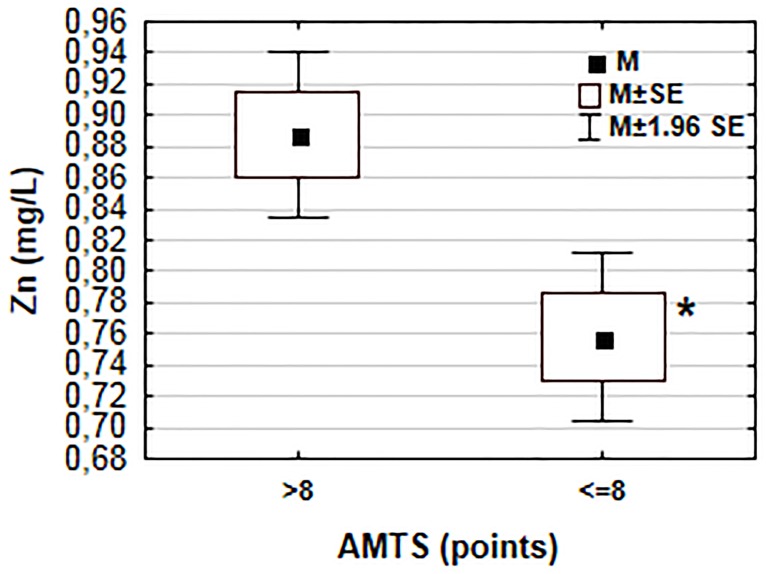
Zinc levels in groups with normal cognitive function and with memory impairment. Asterisk denotes statistically significant difference in serum zinc concentration in the two groups obtained from the Student’s t-test (* p = 0.001). M—mean, SE—standard error

**Fig 3:**
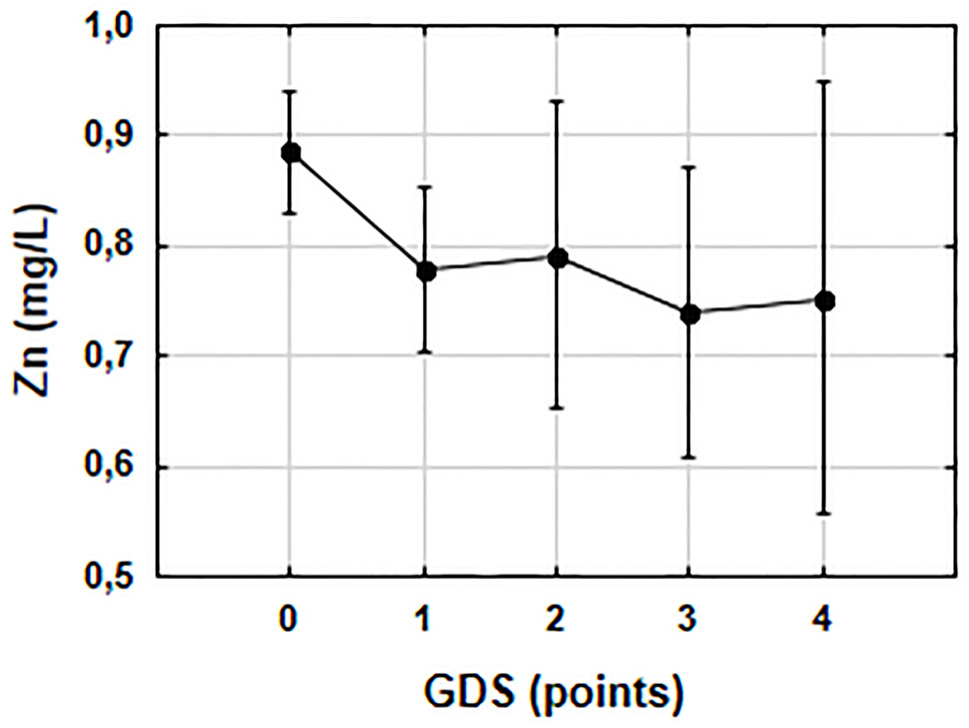
Association between serum zinc concentration and intensity of depression. The results are obtained in ANOVA analysis of variance: F(4,95) = 2.1482, p = 0.081. Vertical bars represent the 0.95 confidence intervals.

Serum Zn concentrations were higher (p = 0.001) in subjects with unimpaired cognitive function (0.89 ± 0.20 mg/L) compared to subjects with memory impairment (0.76±0.19 mg/L) (Fig. 2). Participants showing signs of depression had lower (p = 0.005) serum Zn concentration (0.77±0.17 mg/L) than those without depression (0.89±0.22 mg/L), but there was no association (p = 0.081) between serum Zn concentration and the intensity of depression as shown by the ANOVA test (Fig. 3).

Backward multiple regression analysis was performed, in which the insignificant independent variables were removed one by one i.e. number of medications, BMI, FS, number of diseases, ADL, SRH, age. The obtained results showed that the values of AMTS and GDS together explain 14% of the variation in serum Zn concentration (Table 4).

**Table 4:** The variability in serum zinc concentration depending on the tested parameters.

Variables	β coefficient (SE)	Significance level	Model R^2^
AMTS	0.3030 (0.0934)	0.019	0.14
GDS	-0.2416 (0.0935)	0.043	

The results are obtained by the backward multiple regression analysis (F(2,97) = 8.723 p<0.001, estimation SE: 0.18925), SE—standard error

## Discussion

Data on Zn status among older residents of nursing homes are scarce. To the best of our knowledge, this is the first study that attempts to find a relationship between the Zn status and the mental and physical health in that population.

Plasma or serum Zn concentration is the most commonly and frequently used index for evaluating the probability of Zn deficiency [30]. In the present study, the mean serum Zn concentrations in men and women were 0.84±0.22 mg/L and 0.82±0.19 mg/L, respectively. Our results are in line with other studies i.e. the NHANES II study (1976–1980) [31], which reported 0.87 mg/L and 0.82mg/L, and the AREDS study, reporting 0.85 mg/L and 0.82 mg/L [32]. Furthermore, our obtained values are very close to those reported in Rancho Bernardo study [33] where the mean serum Zn concentration in men was 0.83 mg/L and are also similar to values observed in women from the SU.VI.MAX study, 0.84 mg/L [34]. Our results are slightly higher than the values determined among postmenopausal women in France, 0.80 mg/L [35] or in the United States, 0.75 mgl/L [36].

The percentage of subjects with serum Zn concentration below 0.7mg/L, which is usually considered as the cut-off level for Zn deficiency [18], was 28%. Our findings are comparable with studies carried out in hospitalised elderly people, in which 28% [11] or 38% [12] had plasma Zn concentration below the reference range. This high prevalence of Zn deficiency in nursing home residents is very different from data observed in free-living older population, in whom the reported prevalence rate is 5–6% among healthy subjects participating in the European study ZENITH, 5% in a study in Boston and 3–4% in the Euronut-Seneca European-based study [34, 37].

We observed an age-related change in Zn status (r = -0.28; p = 0.005), which is not in line with the findings of Del Corso et al. [38] but confirms the data by Ravaglia et al. [39]. We found that the prevalence rate of Zn deficiency was 2.5-fold higher among the oldest participants (>80 years) than among persons younger than 70 years.

In our study population we did not find gender differences in Zn status, which is consistent with results of several other studies [40–42].

Grønli et al. [43] shows that Zn deficiency is very common among psychogeriatric patients and it is more prominent in patients suffering from psychiatric disorders other than depression. To our knowledge, no previous studies examined Zn status in nursing home residents with relation to memory function. In our present research, we observed reduced serum Zn concentration in people with memory impairment what indicates that Zn status correlates with cognitive functions.

The relatively high number of individuals with symptoms of depression (48%) revealed in the GDS test is in concordance with the literature [1, 3], however, it should be noted that only 6% of the examined individuals had a documented diagnosis of depression. These findings indicate that GDS test should be routinely performed among nursing home residents since untreated depression negatively affects the quality of life and well-being [44] and can increase mortality [45]. Additionally, in our study we found that older people without symptoms of depression had more athletic body type in comparison to the subjects with depression (p<0.001). Despite growing evidence that depression is a problem, especially in the institutionalised elderly persons, little attention is paid to this issue [3]. In the present study, we found a correlation between the serum Zn concentration and the emotional status—measured by GDS, and almost 2-fold higher prevalence of Zn deficiency in persons with symptoms of depression. This observation is in contrast to the studies by Siwek et al. [46] and Maes et al. [47], but is in line with other studies [48, 49], in which the authors detected a significant correlation. Additionally, a more detailed statistical analysis of our data showed that the magnitude of serum Zn decline did not reflect the severity of the depression. Generally, we found that the residents with depressive symptoms had lower serum Zn concentration (p = 0.005). Similar dependence was observed in clinical studies among patients diagnosed with depression when compared to healthy individuals [47, 49–51].

As the highest Zn concentration in mice was found in the hippocampus region [52], this region seems to be most vulnerable to Zn deficiency [53, 54]. It is this region of the brain which plays a critical role in memory, learning and neurogenesis [54]. In the present study we detected lowered serum Zn concentrations in subjects with cognitive impairment and signs of depression, and found that the values of AMTS and GDS together explain 14% of the variation in concentration of serum Zn. Moreover, a study performed among the community-dwelling elderly persons shows a possible beneficial effect of Zn use in reducing cognitive decline [55]. However, the observed lowered Zn status in older people with cognitive dysfunction requires further investigation.

## Conclusions

Nursing home residents seem to have low Zn status that correlates with their mental health. Their low FS is associated with mental health deterioration and obesity. Our findings highlight the desirability of monitoring the concentration of Zn in serum of older people.
